# Association between Food for Life, a Whole Setting Healthy and Sustainable Food Programme, and Primary School Children’s Consumption of Fruit and Vegetables: A Cross-Sectional Study in England

**DOI:** 10.3390/ijerph14060639

**Published:** 2017-06-14

**Authors:** Mat Jones, Hannah Pitt, Liz Oxford, Issy Bray, Richard Kimberlee, Judy Orme

**Affiliations:** 1 Public Health and Wellbeing Research Group, University of the West of England (UWE), Bristol BS16 1QY, UK; liz@oxf.co.uk (L.O.); issy.bray@uwe.ac.uk (I.B.); richard.kimberlee@uwe.ac.uk (R.K.); judy.orme@uwe.ac.uk (J.O.); 2 Sustainable Places Research Institute, Cardiff University, Cardiff CF10 3BA, UK; PittH2@cardiff.ac.uk

**Keywords:** fruit and vegetables, diet, primary school children, sustainable food

## Abstract

The promotion of dietary health is a public health priority in England and in other countries. Research shows that the majority of children do not consume the recommended amount of fruit and vegetables (F&V). There has been relatively little research on the impact of programmes, such as Food for Life, that (a) integrate action on nutrition and food sustainability issues, and (b) are delivered as commissions in a local authority area. The study sought to assess pupil F&V in schools engaged with the Food for Life (FFL) programme. The design was a cross-sectional study comparing pupils in FFL engaged (n = 24) and non-engaged (n = 23) schools. A total of 2411 pupils aged 8–10 completed a validated self-report questionnaire. After adjusting for confounders, pupils in schools engaged with FFL consumed significantly more servings of F&V compared to pupils in comparison schools (M = 2.03/1.54, *p* < 0.001). Pupils in FFL schools were twice as likely to eat five or more portions of F&V per day (Odds Ratio = 2.07, *p* < 0.001, Confidence Interval = 1.54, 2.77). Total F&V consumption was significantly higher (*p* < 0.05) amongst pupils in schools with a higher level FFL award. Whilst limitations include possible residual confounding, the study suggests primary school engagement with the FFL programme may be an effective way of improving children’s dietary health.

## 1. Introduction

The promotion of healthy child weight and dietary health is a national public health priority in England [1] and in other countries. Evidence shows that fruit and vegetable consumption is an important part of a healthy diet, protects against diet-related disease, and contributes towards healthy weight [2,3,4,5,6,7]. Food-related ill health is responsible for about 10% of deaths and illness, costing the National Health Service about £6 billion annually in the UK [8]. The vast majority of this burden is due to unhealthy diet. Cross-sectional population surveys have shown that the majority of children do not consume the recommended amount of fruit and vegetables [9]. According to a recent national survey [9], only 16% of boys and 17% of girls consume five or more portions of fruit and vegetables a day in England. The same survey also reports children 8–10 years old eat an average of 2.55 portions of fruit and vegetables a day, with the mean number of portions declining from the highest to lowest income quintile [9]. 

Dietary habits acquired in childhood tend to be maintained into adulthood [10,11]. Schools are important for influencing the dietary behaviour of children given that children consume a significant proportion of their diet and develop many nutrition behaviours in this environment [12]. Initiatives in schools also have the potential to reach large and diverse populations and are therefore an obvious focus for universal and equitable public health strategies. A wide variety of interventions have been directed at promoting consumption of fruit and vegetables in schools [13]. Interventions, building on the WHO’s influential Whole Settings model [14], the Whole School Approach [15], and the Health Promoting Schools framework [16], include several components that are intended to generate an effect through interdependent and systemic actions [17]. Van Cauwenberghe et al.’s [18] systematic review of studies in the European Union found evidence of effectiveness of such multi-component programmes in promoting a healthy diet in school-aged children, although a subsequent review found that the evidence is less clear [13]. This work suggests that further evaluative research is needed on whole setting programmes that employ innovative components and design characteristics. The focus of the present study is a scheme that combines a focus on dietary health with wider aspects of food and sustainability. While there is research on the role of specific aspects of food sustainability, such as the role of organic food policies supporting a healthier school food environment [19,20] or school meals as an integrative learning platform for healthy and sustainable food behaviour [21], less is reported on whole setting healthy and sustainable food programmes. The present study focuses on one such programme entitled Food for Life. The aim of the study was to examine the association between primary school engagement in the Food for Life programme and the consumption of fruit and vegetables by children aged 8–10 years. The objectives of the study were (1) to assess fruit and vegetable intake for pupils in schools engaged with Food for Life and for pupils in similar schools not engaged in the programme; and (2) to assess fruit and vegetable intake amongst pupils in schools with different levels of Food for Life awards. A subsidiary objective of the study was to explore further individual and school level variables that contextualise and potentially interact with the association between the programme and fruit and vegetable consumption in pupils.

## 2. Methods

### 2.1. The Food for Life Programme

The focus of the present study is the Food for Life programme. This is a whole school setting multi-component intervention delivered by national charities in England and Wales, with a related scheme in Scotland [22]. The main elements are described in Box 1 and further details are available at: www.foodforlife.org.uk/schools. The programme is organised around the thematic areas of (1) “food education”; (2) “food and catering quality”; (3) “food leadership and school food culture”; and (4) “community and partnerships”. Each theme links to criteria to create a comprehensive framework for changing food culture in schools. Schools that demonstrate meeting a set of criteria are eligible for Food for Life awards graded bronze, silver, and gold.

Box 1 **The Food for Life Programme.**

In the Food for Life programme, schools work towards bronze, silver, and gold mark awards based upon criteria grouped in relation to four programme themes:

**(1) Food education**

Food for Life provides teacher manuals, lesson plans, and project activity packs covering food origins and environmental aspects of farming, growing in school, cooking with unprocessed fruit and vegetables, and sustainably sourced ingredients. Food for Life staff provided guidance on how to integrate these educational resources into the school curriculum such that food sustainability issues would be addressed as a regular element of lessons. Training for school staff covers skills for food growing, cooking, and food based preparation using sustainably sourced ingredients. Food for Life staff advise developing a school garden area, whole-class cookery facilities, and educational links with food producers such as farms and community gardens.

**(2) Food and catering quality**

This component focuses on school food procurement and standards. Food for Life staff deliver training and support for catering teams (cooks and food procurement staff) to make greater use of sustainable food in school meals. Food for Life interprets sustainable foods to include: in-season produce, high animal welfare standards meat, free range eggs, marine conservation certified fish, locally sourced produce, Fair Trade certified produce, produce from a certified organic source, and diets high in fruits and vegetables. All such ingredients are used in menus that comply with or exceed national guidelines on healthy lunch menus.

**(3) Food leadership and school food quality**

This component provides the basis for coordinating the whole school approach. Schools are supported to create a food action group consisting of student representatives, lead school staff and caterers, and parents or other community members. This group sets up consultations with students, parents, staff, and the wider community to identify improvements in all aspects of food in school. As an outcome of this consultation, the group develops a school food policy and action plan that provide reference points for improving the provision of healthier foods including an emphasis on sustainability and wider engagement with food producers and the local community.

**(4) Community and partnerships**

This component establishes formal engagement with parents by means of consultation questionnaires and interactive meetings. This covers strategies for promoting fruit, vegetables, and sustainability issues in school at lunch time, break times, lessons, and after school groups. Parents are provided with written information on the aims of the programme, ideas for using healthy and sustainably sourced ingredients in home cooking projects with children, and ideas for growing fruit and vegetables at home. Parents and wider community members are invited to take part or actively deliver Food for Life-related school activities such as cooking clubs, farm visits, and harvest celebrations.

A central thread that links the different components of the programme is the relationship between dietary health and sustainable food systems. Thus, educational cooking includes learning about using locally grown fresh produce and the environmental aspects of food origins. School cooks develop menus with reduced meat content and make greater use of fresh and minimally processed foods, including fruit and vegetables. School caterers shift their procurement to suppliers that meet higher ethical or welfare standards, and source ingredients from local sources, including, when available, their school garden.

All schools in England and Wales can enrol with the Food for Life scheme and make use of resources (online and print) to support them to implement the programme. By the end of 2015, 5208 schools had enrolled with the programme, of which 1087 had obtained a Food for Life award. The present study focuses on schools that are eligible for a greater level of support offered as part of a locally authority area-based scheme. This is where local government authorities, usually through public health departments, have commissioned Food for Life to deliver additional training, technical advice, and capacity building activities to eligible schools. Food for Life local programme coordinators, alongside national programme experts, deliver these services to teaching staff, school caterers and cooks in clusters of schools. These networks are intended to have an important role in the transfer of best practice between schools and caterers, and to help broker partnership support from, for example, local food suppliers and voluntary groups. The first development phase (2007–2012) of Food for Life found that the programme was associated with a positive impact on fruit and vegetable consumption for children in primary schools [23]. However, this was based upon an intensive model of support with individual Food for Life schools selected to act as national flagships for the programme. It is important to understand the potential effects of the more recent development of the programme (2013 onwards) as it rolls out as a less intensively resourced and area-based initiative.

### 2.2. Study Design and Sampling Strategy

The research followed a cross-sectional design and compared pupils in schools engaged with Food for Life with pupils in schools not engaged with the programme. The study followed a similar approach developed by Keyte et al. in a local authority evaluation of the National Healthy School Programme [24]. The intention was to recruit five Food for Life schools and five Comparison schools in each of five local authority areas with a Food for Life local commission that had been running for at least 24 months. The target respondents were children aged 8–10 years in school Years 4 and 5. Keyte et al. [24]’s study, working with a similar questionnaire tool, target population, and outcome measures, estimated that a sample of 50 children in each school recruited to the study would provide acceptable levels of precision for measuring the associations required in this study. 

Selection and recruitment of schools followed a systematic process. Local programme managers in each local authority commissioned area were asked to provide a list of all ‘Food for Life schools’ defined as those that met at least four of the following criteria: (1) delivering cooking, growing, food sustainability, and/or farm visit activities for pupils within class teaching within the last year; (2) consulting with pupils and/or parents about school food and catering quality at least termly; (3) having a food policy and action plan written or revised within the last 3 years; (4) participating in at least one Food for Life community and partnership training session within the last year; (5) having a designated Food for Life co-ordinator; (6) holding a current Food for Life award (bronze, silver, or gold). In almost all cases the clearest indicator of engagement was a current Food for Life award. In two of the five local authority areas, local programme managers nominated schools that had not achieved an award, but had been a focus for engagement in the local commission contract and achieved other stated criteria. 

From this group of Food for Life schools, five were selected by list number for each local commission area. A letter was sent to the headteacher of each school by email, detailing the study and requesting participation of one class from both Years 4 and 5. Where a school declined, the next school listed was invited to participate. Comparison schools were selected from a list of all remaining primary schools in the local authority by finding a best match in terms of (a) national tertile for school size, as measured by number of pupils on the school roll; (b) national quintile for the proportion of pupils with free school meal eligibility (FSME). FSME was used as a proxy measure of socio-economic status [25]. Despite a number of limitations, in UK educational research FSME is widely used as a proxy for family socio-economic status; a predictor for individual and school level attainment at Key Stage 2; and is linked to other school-level variables such as those of special needs, first language, living in care, and school mobility [26,27]. FSME has also been assessed as having a number of advantages over area-based measures, such the Index of Multiple Deprivation, as a parameter by which to compare schools [27]. Where multiple similar matches were available, a school was selected using an online number randomizer (https://www.randomizer.org). Sampling therefore followed a process that sought to reduce sources of selection bias and optimise the match between two groups. Headteachers (or a nominee) who consented to participate completed a brief questionnaire regarding their school’s Food for Life related activity in order to confirm the school’s engagement in the Food for Life programme against the criteria.

### 2.3. Data Collection with Pupils

Data collection in participating schools took place in one of two waves, either October–November 2014 or February–April 2015, and took place on school days between Tuesday to Friday. The researcher arranged a time and date to visit each school. During each class visit a checklist was used to ensure a consistent approach during questionnaire completion. This had been developed following piloting and lunchtime observations with Year 4 and 5 pupils in four schools not included in this study. Pupils were eligible for the study if they were aged between 8–10 years and in school Years 4 and 5. Before completing the survey, pupils were asked whether they were happy to complete the questionnaire or whether they would prefer to do an alternative activity, such as reading a book. The questionnaires were completed as a whole class activity with the teacher, teaching assistant, and researcher present. Pupils were advised that they could ask for help reading the questions, or for clarification of their meaning at any time, and individual pupils received additional support as necessary. The questionnaire was completed, without exception, within 30 min for each class visit. Of total eligible pupils, 3% did not complete the questionnaire due to class absence or withdrawal of consent, giving a pupil response rate of 97%.

### 2.4. Questionnaire 

The Day in the Life Questionnaire (DILQ) is a validated questionnaire, utilising the 24 h recall method of collecting dietary information, specifically designed to measure fruit and vegetable consumption in primary school aged children [28]. DILQ is identified as a suitable tool in Public Health England’s Standard Evaluation Framework for Dietary Interventions [29]. The questionnaire asks the respondent to recall everything that they had done the day before and, to minimise recall bias, does not focus solely on food and drink consumed. Respondents are asked to list all items of food and drink consumed and, to aid recall, draw all items for main meals.

### 2.5. Summary of Ethical Issues

Ethical approval for this study was obtained in May 2014 through the Research Ethics Committee of the Faculty of Health and Applied Sciences, UWE [Ref: HAS/14/05/79]. Headteachers (or a nominee) were assured school anonymity and asked to provide informed written consent. Headteachers were provided with the following information to distribute in advance to parents/guardians of children: a letter of introduction, copy of the questionnaire, information sheet, and an opt-out form. Before taking part, pupils were advised about the confidentiality and anonymity of the questionnaire, publication of the research, and asked whether they were happy to complete the questionnaire or whether they would prefer to do an alternative activity, such as reading a book. 

### 2.6. Data Processing and Analysis 

Data written on the questionnaires was coded and then inputted manually into Excel and exported to SPSS, Version 20 (IBM, 2015, New York, NY, USA). The following decisions were made: a total of 45 respondents were excluded due to being either outside the 8 to 10 year age bracket or providing a largely incomplete questionnaire; for 26 respondents, missing data for gender and age were imputed using the rule of replacing the missing data with the modal value for the school of the respondent. The latter approach was used because the order of questionnaire retrieval followed the grouping of pupils in the classroom. Research shows that pupil grouping tends to be clustered by age and gender in UK primary school classroom settings [30].

Following the DILQ guidance, all discrete items fruit and vegetables were recorded (for coding details, see Edmunds and Ziebland [28]). We recorded up to one serving of fruit juice although, given the potential for pupils to confuse fruit juice with added sugar fruit drinks, these data were treated and reported on separately from the main analysis. The DILQ does not at the point of coding attempt to quantify the consumption of fruit and vegetables in terms of portion size. Rather, its main utility is in determining differences in fruit and vegetable intake at group level [28]. In this study, we interpreted counts of fruit and vegetables as ‘servings’ at the point of reporting following the convention of other studies [31]. When interpreted as total daily servings, the results might be considered conservative because they do not include some dietary sources of fruit and vegetables, for example, as a constituent of composite foods. 

Coders and inputters were blinded to condition of the school. A 5% random sample was inter-rater reliability tested and found a good agreement (κ = 64, *p* < 0.001) [32]. The assessment of outcome variables was achieved using an Independent Samples T test, Pearson’s Chi Squared test, or Kriskal-Wallis H test where appropriate. Binary logistic regression was used, where indicated, to determine odds ratios after controlling for potential confounders. All reported *p* values are from two-sided statistical tests and differences with *p* ≤ 0·05 were considered significant. The dataset generated during and analysed during the current study are available in the figshare repository [33]. 

### 2.7. Characteristics of Participating Schools and Pupils

Table 1 shows that the five local authority study settings included both rural, urban, and mixed areas. Of those approached, 72.7% (n = 24/33) of Food for Life schools and 41.8% (n = 23/55) of Comparison schools approached agreed to take part in the study. Further details of the study schools are provided in Table 1. Table 2 shows that there were no significant differences in the size of school, that is, the total number of pupils on roll, or percentage for Free School Meal Eligibility (FSME) between Food for Life and Comparison schools, suggesting the groups were matched with reference to these parameters. The mean FSME for Food for Life schools and Comparison schools was 18.9% (SD 13.6) and 17.2% (SD 13.0), respectively. In addition, there were no significant differences between the local authority area groups in terms of school size or FSME.

The total number of children included in the study was 2411. All the children were in Year 4 or 5. The age range was 8 to 10 years old and with a similar proportion of boys and girls (Table 3). FSME% was used as a proxy measure for socio-economic status. The sample of pupils broadly reflected the national distribution of FSME quintiles, although there were fewer in the second FSME quintile (11.8%).

## 3. Results

### 3.1. Fruit and Vegetable Consumption of Pupils

#### All Schools

Table 4 shows that the mean number of servings self-reported for ‘total fruit and vegetables’ was 1.80. More than half (59%) of fruit and vegetables were consumed in school. Fruit made up the greater share (59%) of total fruit and vegetables in reported consumption.

The mean number of fruit and vegetable servings consumed in this survey was less than the mean of 2.55 portions recently reported nationally [9]. This is likely to be due to the measurement characteristics of the DILQ tool that does not take into account fruit juice and fruit and vegetables in composite foods. If fruit juice is included in the analysis, up to a maximum of one serving, the mean fruit and vegetable consumption increases from 1.80 to 2.37 servings. This is closer to the national survey average. 

National guidelines recommend that five plus portions of fruit and vegetables are consumed each day. Table 5 shows that, using the unadjusted DILQ servings, 9.5% (n = 230) of pupils reported eating five plus servings of fruit and vegetables per day. Additionally, 28.4% (n = 684) reported eating no fruit or vegetables at all during the preceding day. Supplementary analysis showed that 51.7% of children reported eating no fruit or vegetables before school (at breakfast or before arrival) or after school (in the period from the end of school to an evening meal, at an evening meal, or during the evening/before bed).

We tested the association between the mean number of fruit and vegetable servings consumed and other variables in order to understand their potential interactions with the main study objectives. Age was not significantly associated with fruit and vegetable consumption (*p* = 0.082). Girls reported eating significantly more fruit and vegetables than boys (girls: M = 2.10; boys: M = 1.52; *p* < 0.001). Fruit and vegetable consumption was associated with FSME% (*p* < 0.001): pupils in schools with a higher FSME% consumed less fruit and vegetables than those in schools with a lower FSME%. The mean number of fruit and vegetable servings reported varied between local authority areas. It was highest in local authority commission B (M = 2.10) and lowest in local authority commission D (M = 1.50, *p* = 0.003) (Data not reported in a separate table).

### 3.2. Food for Life Schools and Comparison Schools

Pupils in Food for Life schools were significantly more likely to consume more servings of fruit and vegetables than pupils in Comparison schools: for total fruit and vegetable consumption, pupils in Food for Life schools reported consuming nearly a third (31.8%) more than pupils in Comparison schools (M = 2.03/1.54; *p <* 0.001). This significant difference is also evident for all sub-measures for fruit and vegetable consumption, apart from vegetable consumption out of school (see Table 4).

There was also a difference in the number of pupils in Food for Life and Comparison schools reporting five plus servings of fruit and vegetables; 12.3% of pupils consumed five or more servings in Food for Life schools and 6.5% of pupils consumed five or more servings in Comparison schools (Table 5). In addition, 23.4% of pupils in Food for Life schools and 33.9% of pupils in Comparison schools were recorded as eating no fruit and vegetables. Further analysis across the course of the day showed that 49.6% of pupils in Food for Life schools reported eating no fruit and vegetables at home, whereas this figure was 54.4% for pupils in Comparison schools. 

Pupils were grouped into categories of (a) five or more servings of fruit and vegetable consumed and less than five servings, and (b) 2.55 servings or more of fruit and vegetables consumed and less than 2.55 servings. As shown in Table 6, the association previously seen between fruit and vegetable intake and engagement with Food for Life persisted in this analysis. 

Using binary logistic regression, we sought to test the effect of Food for Life on pupil consumption of five or more servings of fruit and vegetable per day. The model controlled for FSME, gender, and local authority area as potential confounders. We found that pupils in schools engaged with the Food for Life programme had double the odds of eating five or more servings of fruit and vegetables per day compared to pupils in Comparison schools (OR (Odds Ratio) = 2.07; *p* < 0.001; CI (Confidence Interval) 1.54, 2.77).

National survey data reports that pupils aged 8–10 years eat an average of 2.55 portions of fruit and vegetables per day [9]. After adjustment for FSME and gender, the odds of reporting eating 2.55 or more servings of fruit and vegetables a day were 60% higher for pupils in Food for Life schools (OR = 1.66; *p* < 0.001; CI = 1.37, 2.00).

### 3.3. Schools and Food for Life Award Status

This section of the findings reports on the relationship between the main outcome and the level of Food for Life award that schools achieved. Preliminary analysis found that silver Food for Life award schools were over twice as likely to eat five plus portions of fruit and vegetables compared to pupils in schools with no Food for Life award (15.6% and 6.7%, respectively). Pupils in schools with no Food for Life award were almost twice as likely to consume no fruit or vegetables compared to pupils in silver Food for Life award schools (34.1% and 18.1%, respectively). Approximately one and a half times more pupils in Food for Life silver award schools ate five plus portions or more a day of fruit and vegetables compared to those in Food for Life bronze award schools (15.6% an 10.3%, respectively).

Table 7 shows the means and standard deviations for each of the three groups of (1) schools with no award; (2) bronze award schools; and (3) silver award schools with respect to total fruit and vegetable consumption, and the other sub-measures of fruit and vegetable consumption. A Kruskal-Wallis H test was conducted to compare the effect of Food for Life award status on pupil total fruit and vegetable consumption. The result showed that there was a statistically significant difference in total fruit and vegetable consumption between Food for Life award status of schools, χ^2^(2) = 51.242, *p* < 0.001, with a mean rank score of 1116.31 for no Food for Life award schools, 1281.21 for Food for Life bronze award schools, and 1346.82 for Food for Life silver award schools. Post hoc comparisons were conducted to determine which pairs differed significantly. Table 7 shows the results found that pupils in silver award schools consumed more fruit and vegetables (M = 2.18, SD = 1.20) than those in bronze award schools (M = 1.97, SD = 1.86), who in turn consumed more than those in schools with no award (M = 1.57, SD = 1.72), Adj.Sig. *p* < 0.05 for all pairs. A similar test procedure was conducted for selected sub-measures. A test of fruit and vegetable consumption in school found the same pattern of results, Adj.Sig. *p* < 0.05 for all pairs. Fruit and vegetable consumption out of school was higher for pupils in schools with any Food for Life award than in schools with no award (Adj.Sig. *p* < 0.05), but there was no statistical difference between pupils in silver award schools and those in bronze award schools, Adj.Sig. *p* = 0.965.

## 4. Discussion

### 4.1. Fruit and Vegetable Consumption

This study found that the mean number of servings of fruit and vegetables self-reported by Year 4 and 5 pupils (aged 8–10 years) in Food for Life engaged schools was significantly higher than the number of servings reported by pupils in Comparison schools. Whilst recognising the limitations of the Day in the Life Questionnaire methodology in assuming that fruit and vegetable servings are equivalent with portion sizes, it is possible that this difference could be approximately 0.5 portion or 40 grams difference between the two groups. This finding is consistent with a recent meta-analysis of school-based interventions that found an improvement of 0.25 portions of fruit and vegetables if fruit juice was excluded and 0.32 portions if fruit juice was included [34]. 

For all pupils, mean daily fruit and vegetable consumption was well below the public health five-a-day guidelines, although this is consistent with evidence from other research studies with this age group in Europe, the USA, and Australia [35,36,37]. The study found that a high proportion (28.4%) of participants reported eating no fruit or vegetables at all during the 24 h prior to the survey. This proportion was lower in Food for Life schools (23.4%) than in Comparison schools (33.9%). The wide gap between guidance and practice underscores the importance of improving dietary behaviours of children. It highlights the importance of the school environment given that, for many children, there are limited or no opportunities to eat fruit and vegetables at home. In this context, evidence of a difference in diet is notable given that fruit and vegetable consumption in Food for Life schools was not only higher within school time, it was also higher at home. This finding is consistent with the Food for Life programme aspiration to have an impact that spills over from the school to the home, and suggests an extension of the programme’s impact into the wider community.

As a whole setting-based model, the Food for Life programme has a range of processes and mechanisms that may contribute towards a positive impact on dietary behaviour. The focus on freshly prepared and minimally processed foods, including fruit and vegetables, in Food for Life school meal standards, combined with measures to promote school meal take up (as opposed to packed lunches from home) appears to have a plausible, direct impact. More systemically, the scheme aims to coordinate the role of educational and food catering activities, staff training, and stakeholder participation in multiple areas of school life. Measures seeking to promote both the nutritional health and the sustainability aspects of food may interact to produce effects greater than those that would occur through uncoordinated action. The exchange of best practice between school and catering staff within local geographical areas represents a further mechanism for driving change. Positive outcomes for the programme were more consistent in some local authority areas than others in this study than others. This highlights the need to build upon formal learning of what works in each area and to enhance programme elements that are likely to have the greatest impact. 

The Food for Life award framework, from bronze to silver to gold, aims to promote incremental changes across a wide range of food related activities. Although the potential of this model is widely recognised in the literature on healthy school settings [38], evidence on the effects of specific programme mechanisms is less clear [18]. The clearest evidence of an association between mechanisms and outcomes was with respect to the award status of schools; the study found that pupils in Food for Life silver award schools ate more fruit and vegetables than those in Food for Life bronze schools or schools without an award, although the differences between bronze and silver award status were clearer for fruit and vegetable consumption in school than out of school. At the time of undertaking the research, only a small number of schools, nationally, had achieved the Food for Life gold award, and none took part in the present study. Outcomes for schools achieving this higher level of award could be a focus for research in the future. 

### 4.2. Study Strengths and Limitations

Strengths of the study include the large number of schools recruited to the study in five local authority areas, the large pupil sample size, the measures taken to control for confounders and self-selection in the school recruitment process, and the use of a well-recognised validated tool for dietary assessment with this age group [28,29]. 

A number of study limitations need to be recognised. There was possible residual confounding by socio-economic factors. For each local authority area, we were not able to able to achieve complete matches for each Food for Life school in terms of the FSME quintile and the number of students on roll. Nevertheless FSME% at school level was adjusted for in our analyses. Other indicators could have been drawn upon, such as those linked to attainment and local area deprivation, to assist with matching Food for Life and Comparison schools. However, FSME was used as a key indicator due to its widespread use regarding issues of equity in educational policy and practice [26]. The sampling approach may also have been affected by a selection bias: schools that agreed to participate were perhaps more highly engaged in healthy food related activities. However, it is not clear how this would have systemically affected two groups in different ways. 

Seasonality may have had an effect on the study, given that surveys for two local authority areas had to be conducted in two waves: autumn and spring during the school year. However, initial piloting that included repeat surveys over two seasons identified no evidence of seasonality.

Whilst it is a validated tool, the DILQ does not measure fruit and vegetables within composite foods, such as pizzas or pies. The explanation given is that interventions that encourage an increase in fruit and vegetable consumption do not usually include composite foods [28]. It would also be too difficult to estimate their contribution to the diet [39]. In the Health Survey for England, fruit and vegetables are included only if they are a main constituent of the food such as stewed fruit or vegetable curry [9].

Composite foods could be potentially significant in the context of the Food for Life programme, given that the initiative includes a focus on including fruit and vegetables as part of composite dishes in school meals. We were not able to directly assess the contribution of these dishes towards student diets. Further research is needed to assess the feasibility of using an adapted version of the DILQ tool for the assessment of composite dishes, or to validate an alternate tool appropriate to the Food for Life programme context and have access to the recipes used in school meals.

It would have been desirable to undertake further dietary assessment through, for example, school mealtime observations and analysis of food plate waste [28], however, this would have involved a considerably more intensive programme of research that was beyond the resources available to the team. The study did not assess consumption of dietary components apart from fruit and vegetables, such as sweets or soft drinks. Although an exploratory and inconclusive assessment was made of sweet snack and savoury (salty) snack consumption with a subset of the data, these dietary aspects fell outside the original research protocol and are not reported on in the current article.

### 4.3. Policy and Practice Implications

There are a number of policy and practice implications arising from this study. The design of school food programmes might incorporate components that have a focus on sustainable food issues as an additional and complementary focus on the dietary health aspects of food. Schools and partner agencies may seek strategic support from specialist programme agencies to enhance their implementation of award schemes such as Food for Life, although further research is warranted on the link between implementation and health outcomes. Primary school programmes delivered on an area-basis, such as across a local authority area, may offer the basis for reaching large pupil populations.

## 5. Conclusions

This is the first study of Food for Life, when commissioned as a local authority area-based programme, to evaluate dietary behaviour using a cross-sectional school-matched comparison approach. Whilst limitations of the study design and its implementation need to be recognised, the study found evidence of a positive impact of a multicomponent school settings-based programme. Given the challenges of promoting nutritional and food change at a population level, Food for Life appears to have a role as part of an area-based approach to coordinate dietary improvements through schools and catering agencies. For schools participating in the programme, progression from bronze towards silver Food for Life award status appears to be an important part of the process in improving dietary outcomes. 

## Figures and Tables

**Table 1:** Characteristics of the study population by local authority area commission.

Local Authority Commission	Local Authority Urban-Rural Description	Totals for Primary Schools in Local Authority			Food for Life Schools			Comparison Schools			Total Pupils
		Food for Life Schools in LA (n)	Schools not Engaged in Food for Life in LA (n)	Total Primary Schools in LA (n)	Total Schools Contacted (n)	Total Study Schools (n)	Pupils (n)	Total Schools Contacted (n)	Schools (n)	Pupils (n)	Pupils (n)
A	Urban conurbation	44	45	89	7	5	296	14	3	132	428
B	Mixed: small town/rural	24	142	166	7	5	267	11	5	288	555
C	Urban conurbation	38	102	140	6	5	258	12	5	229	487
D	Mixed: small town/rural	26	42	68	8	5	215	10	5	230	445
E	Mixed: large town/rural	18	94	112	5	4	229	8	5	267	496
Total		150	425	575	33	24	1265	55	23	1146	2411

**Table 2:** Characteristics of school sizes (pupil roll) and school level Free School Meal Eligibility (FSME).

	No. Pupils on School Roll			Free School Meal Eligibility FSME%		
	Mean No.	Min/Max (Range)	Standard Deviation	Mean FSME%	Min/Max (Range)	Standard Deviation
By Status						
Food for Life (n = 24)	276	67–618 (551)	131.9	18.9	2.7–46.7 (44.0)	13.6
Comparison (n = 23)	236	110–390 (280)	83.2	17.2	2.7–42.2 (39.5)	13.0
*T* test result	*p* = 0.232			*p* = 0.654		
By Local Authority Commission						
A (n = 8)	275	108–502 (394)	133.1	13.5	3.1–19.9 (16.8)	6.7
B (n = 10)	287	110–618 (508)	152.2	24.2	2.7–45.6 (42.9)	14.1
C (n = 10)	275	174–390 (216)	85.4	23.6	7.1–46.7 (39.6)	16.2
D (n = 10)	256	67–323 (256)	92.3	15.9	2.7–42.2 (39.5)	14.4
E (n = 9)	253	136–361 (225)	73.0	11.6	2.7–23.5 (20.8)	7.1
*T* test result	*p* = 0.380			*p* = 0.113		
Total		37–618 (581)	111.3	18.1	2.7–46.7 (44.0)	13.2

**Table 3:** Characteristics of pupils in the whole study sample (n = 2411).

		Pupils Participating (n)	Pupils Participating (%)
Gender	Boy	1240	51.4
	Girl	1171	48.6
Age	8	762	31.6
	9	1161	48.2
	10	488	20.2
Socio-economic status (FSME quintile) *	Top quintile (41.6%+)	438	18.2
	2nd quintile (25.5–41.5%)	285	11.8
	3rd quintile (15.7–25.4%)	606	25.1
	4th quintile (9.3–15.6%)	484	20.1
	Bottom quintile (0–9.2%)	598	24.8
Attending a school engaged with Food for Life?	Yes	1265	52.5
	No	1146	47.5
Attending a school with Food for Life award?	No award	1293	53.6
	Bronze	632	26.2
	Silver	486	20.2

* Socio-economic status as defined by percentage of free school meal eligibility of school (FSME %). FSME quintiles are calculated nationally by ranking the FSME% data for all schools and then splitting this data into five sub-groups, each representing approximately 20% of all schools.

**Table 4:** Mean number of servings of fruit and/or vegetables consumed by pupils in Food for Life schools and Comparison schools.

Servings	All Schools		Food for Life Schools		Comparison Schools		Significance
	n = 2411		n = 1265		n = 1146		
	Mean	SD	Mean	SD	Mean	SD	*p*
Fruit and vegetables in school	1.07	1.17	1.24	1.22	0.89	1.08	0.000
Fruit in school	0.69	1.01	0.78	1.06	0.59	0.94	0.000
Vegetables in school	0.38	0.60	0.46	0.63	0.30	0.55	0.000
Fruit and vegetables out of school	0.73	0.94	0.79	0.99	0.65	0.88	0.000
Fruit out of school	0.38	0.68	0.44	0.73	0.33	0.62	0.000
Vegetables out of school	0.34	0.59	0.36	0.60	0.32	0.58	0.174
Total fruit and vegetables	1.80	1.83	2.03	1.93	1.54	1.68	0.000
Total fruit	1.07	1.52	1.21	1.61	0.92	1.41	0.000
Total vegetables	0.76	0.94	0.86	0.97	0.65	0.90	0.000
Total fruit and vegetables including one serving juice (max.)	2.37	1.95	2.64	2.04	2.07	1.79	0.000

**Table 5:** Servings of fruit and vegetables consumed by pupils.

Servings	All schools		Food for Life Schools		Comparison Schools	
	n	%	n	%	n	%
0	684	28.4	296	23.4	388	33.9
1	654	27.1	345	27.3	309	27.0
2	396	16.4	214	16.9	182	15.9
3	262	10.9	140	11.1	122	10.6
4	185	7.7	114	9.0	71	6.2
5+	230	9.5	156	12.3	74	6.5
Total	2411	100	1256	100	1146	100

**Table 6:** Numbers of pupils consuming five or more servings and 2.55 or more of fruit and vegetables according to school engagement with Food for Life.

**Fruit and Vegetable Intake**	**Five Servings or More n (%)**	**Less than Five Servings n (%)**	**Significance *p***
All pupils (n = 2411)			
Food for Life schools	156 (12.3%)	1109 (87.7%)	0.000
Comparison schools	74 (6.5%)	1072 (93.5%)	
**Fruit and Vegetable intake**	**2.55 Servings or More n (%)**	**Less than 2.55 Servings n (%)**	**Significance *p***
All pupils (n = 2411)			
Food for Life schools	410 (32.4%)	855 (67.6%)	0.000
Comparison schools	267 (23.3%)	879 (76.7%)	

**Table 7:** Mean number of servings of fruit and/or vegetables consumed by pupils by Food for Life award status.

	No Food for Life Award		Bronze Award		Silver Award		Adjusted Significance
	n = 1293		n = 632		n = 486		
	Mean	SD	Mean	SD	Mean	SD	
Total fruit and vegetables	1.57	1.72	1.97	1.86	2.18	1.20	All pairs: *p* < 0.05
Fruit and vegetables in school	0.91	1.12	1.19	1.17	1.37	1.24	All pairs: *p* < 0.05
Fruit and vegetables out of school	0.66	0.88	0.78	1.01	0.82	1.00	No award vs. FFL award: *p* < 0.05 Bronze vs. silver award: *p =* 0.965

## Supplementary Materials

The dataset generated and analysed for this study is available at figshare https://dx.doi.org/10.6084/m9.figshare.3749457.v1.
